# Complete genomic sequencing of a *Pseudomonas putida* isolate derived from a patient suffering from chronic diarrheal infection in Hangzhou, China

**DOI:** 10.1128/mra.00885-23

**Published:** 2023-12-15

**Authors:** Qin Wang, Sayyed Salman

**Affiliations:** 1 Department of Obstetrics and Gynecology, Key Laboratory of Reproductive Dysfunction Management of Zhejiang Province, Zhejiang Provincial Clinical Research Center for Obstetrics and Gynecology, Sir Run Run Shaw Hospital, School of Medicine, Zhejiang University, Hangzhou, China; 2 Collaborative Innovation Center for Diagnosis and Treatment of Infectious Diseases, State Key Laboratory for Diagnosis and Treatment of Infectious Diseases, the First Affiliated Hospital, College of Medicine, Zhejiang University, Hangzhou, China; The University of Arizona, Tucson, Arizona, USA

**Keywords:** *Pseudomonas putida*, genome analysis

## Abstract

This study presents a comprehensive analysis of the genomic sequence of *Pseudomonas putida* isolate L2890hy, having a 6,733,472 bp chromosome size. This particular strain was obtained from a fecal sample of a patient suffering from acute diarrhea in Hangzhou, China, in 2020.

## ANNOUNCEMENT

*Pseudomonas putida* is a Gram-negative rod-shaped bacterium that belongs to the fluorescent group of pseudomonads. It is usually found on inert surfaces in hospitals and humid places due to its strong tolerance to harsh living conditions and causes nosocomial infections (1). A case of acute diarrhea with *P. putida* is examined in this research. Since 2016, a prospective survey has examined carbapenem-resistant gut colonization and risk variables in hospitalized diarrheal patients (2). A stool sample (1.0 g) was collected from a 71-year-old hospitalized patient with diarrhea in October 2020. The fecal sample was diluted in 5  mL of sterile Brain Heart Infusion Broth and then cultured overnight at 37°C on MacConkey agar with 2 mg/L meropenem. Colonies exhibiting different morphologies were subjected to repeated streaking on MacConkey agar treated with Meropenem to obtain a pure culture. Bacterial species were identified by matrix-assisted laser desorption/ionization time-of-flight mass spectrometry (Bruker, Bremen, Germany) (3).

The genomic DNA was extracted from a culture grown overnight at 37°C using the Gentra Puregene Yeast/Bact kit (Qiagen). The DNA was evaluated by gel electrophoresis, and quality and quantity were checked by a NanoDrop 2000 UV-visible spectrophotometer (Thermo Scientific, USA) and a Qubit version 2.0 fluorometer (Thermo Scientific) (2). The same DNA was then sequenced on Illumina, Novogene Bioinformatics Technology (Beijing, China) and Oxford Nanopore. For the short-read sequencing, DNA was then fragmented by sonication to a size of 350 bp. Then, the sequencing library was generated with the Nextera XT kit (Illumina, USA) and subsequently subjected to sequencing using the Illumina NovaSeq 6000 platform. The A-tailed fragments were subjected to ligation with paired-end adaptors, followed by PCR amplification using a 500-bp insert. After PCR products were purified by the AMPure XP system (USA), library quality was assessed on the Agilent 5400 system (USA) and quantified by QPCR (1.5 nM) by using the default parameters. The DNA library was prepared for long reads using the ligation sequencing Kit (SQK-LSK114). Before library preparation, genomic DNA was not mechanically or enzymatically sheared for long-read sequencing. Raw reads from the PromethION were initially assessed using the MinKNOW software for real-time QC metrics and processed with the Oxford Nanopore Technologies Guppy software (version: 0.17.1).

The short raw read quality statistical analysis was performed using Fastp (version 0.23.1), and the hybrid assembly was conducted via Unicycler version 0.4.7 (4). The sequence was then submitted to the NCBI for annotation and bioinformatics analysis by NCBI Prokaryotic Genome Annotation Pipeline (5, 6), and it was performed using a best-placed reference protein set, and GeneMarkS-2+ acquired antibiotic resistance genes in L2890hy were identified by Res-Finder 4.1. Related metrics are listed in Table 1.

**TABLE 1:** Features of the complete whole-genome sequences of *Pseudomonas putida* strain L2890hy

Feature	Data for strain *Pseudomonas putida* L2890hy
Long read	
Number of reads	146,299
Total bases (bp)	1,355,477,532
Mean read length (bp)	9,265.1
N50 read length (bp)	12,274
Mean read quality	11.3
Short read	
Raw reads	9,958,510
Clean reads	9,902,418
Raw base (G)	1.49
Clean base (G)	1.49
Effective (%)	99.44
Q20 (%)	97.83
Q30 (%)	93.87
GC (%)	60.99
Assembly	
Genome size (bp)	6,733,472
Coverage (X)	201
GC content (%)	61.2
N50 value	6,302,573
L50 value	1
Resistance gene (antimicrobial)	
Chromosome	*aph(3'')-Ib, aph(6)-Id* (aminoglycoside) *sul1, sul2* (sulfonamide)
Plasmid	*ant(2'')-Ia, aadA24, aac(6')-IIa , armA, aac(6')-Il* (aminoglycoside) *qacE* (disinfectant) *blaOXA-246* (beta-lactam) *tet(G)* (tetracycline) *ARR2, ARR-3* (rifampicin) *qnrVC1* (fluoroquinolone) *mph(E)* (macrolide) *msr(E)* (macrolide, lincosamide and streptogramin B) *cmlA1* (phenicol) *sul1* (sulfonamide) *dfrA27* (trimethoprim)

The complete and circular chromosome size of *P. putida* L2890hy is 6,733,472 bp. The GC content was approximately 61.2%, with an *L*_50_ value of 1. Antimicrobial resistance gene analysis showed that the chromosome contained two antimicrobial resistance genes, including the *aph*(3*″*)*-Ib, aph*(*6*)-*Id* (aminoglycoside) and *sul1, sul2* (sulfonamide). The plasmid-finder analyses showed that *P. putida* harbored 10 antibiotic resistance genes on the plasmid, along with the *bla*_OXA-246_ gene conferring resistance to beta-lactam antibiotics.

## Data Availability

The whole-genome sequence of *P. putida* L2890hy has been deposited in the GenBank under accession numbers CP134602 and CP134603, BioProject accession number PRJNA1014128, BioSample accession number SAMN37319580, and SRA number SRX22388373. The version described in this paper is the first version.
